# Effects of Probiotics Supplementation on Risk and Severity of Infections in Athletes: A Systematic Review

**DOI:** 10.3390/ijerph191811534

**Published:** 2022-09-13

**Authors:** Mirella Di Dio, Patrizia Calella, Giuseppe Cerullo, Concetta Paola Pelullo, Valeria Di Onofrio, Francesca Gallè, Giorgio Liguori

**Affiliations:** 1 Department of Movement Sciences and Wellbeing, University of Naples “Parthenope”, 80133 Naples, Italy; 2 Department of Sciences and Technologies, University of Naples “Parthenope”, 80143 Naples, Italy

**Keywords:** athletes, probiotic, diet supplementation, gastrointestinal disorders, upper respiratory tract infections

## Abstract

The aim of this review was to appraise the literature on the effects of probiotics supplementation on gastrointestinal (GI) and upper respiratory tract infection (URTI) risk and prognosis in athletes. The search was conducted using the following electronic databases: MEDLINE (PubMed); Web of Science; Scopus; and SPORTDiscus (EBSCO). According to the PRISMA guidelines, randomized controlled studies performed on healthy athletes with a note dose of probiotics supplementation were considered. From the 2304 articles found, after eliminating reviews and studies on animals and unhealthy subjects and after screening of titles and abstracts, 403 studies were considered eligible. From these, in accordance with the inclusion and exclusion criteria, 16 studies were selected, ten of which concerned endurance athletes. The majority of the studies reported beneficial effects of probiotics in reducing the risk of developing the examined infections or the severity of related symptoms. However, due to the differences in formulations used and populations analyzed in the available studies, further research is needed in this field to achieve stronger and more specific evidence.

## 1. Introduction

Nowadays, it is well established that physical activity (PA) can have beneficial effects on the whole human body and strength its immune defenses. In fact, scientific evidence has established that regular PA is effective in the prevention of various chronic diseases, such as cardiovascular diseases, diabetes, cancer, hypertension, obesity, depression, osteoporosis, and premature death [1,2]. However, the effects of PA can be different depending on its intensity and duration.

In particular, high-intensity PA and strenuous exercise seem to increase intestinal permeability and diminish gut mucus thickness, potentially enabling pathogens/toxins to enter the bloodstream, and have been associated with immunosuppression by decreasing immune cell function, which enhances susceptibility to infections [3,4,5,6,7,8,9].

In fact, during heavy training and competitions, a higher incidence of gastrointestinal (GI) disorders, such as diarrhea and heartburn [7,8], and upper respiratory tract infections (URTIs) can occur [9,10,11]. This is due to acute post-exercise immune breakdowns and chronic suppression of immune factors, dependent on frequent strenuous exercise [12,13]. Therefore, reducing these symptoms in athletes becomes a top priority. Nowadays, probiotic supplement use is promoted in this perspective. The term “probiotic” refers to those microorganisms, which, once ingested in adequate quantities, are able to exercise beneficial functions for the body [14].

Indeed, there is increasing evidence to support the efficacy of probiotic supplementation, alone or in combination with prebiotics, in reducing the number, duration, and severity of acute infectious diarrhea and URTI cases in the general population [15,16,17]. The interest in athletes’ diet supplementation arises from this evidence. Essentially, probiotics would act by moderating the negative effects of acute and chronic intense exercise on the immune system, thus reducing susceptibility to the aforementioned pathologies [15,16,17].

The main aim of this review was to critically appraise the literature on the effects of probiotics supplementation in the prevention of GI and URTI in athletes. The role of probiotics in the reduction of GI and URTI related symptoms was also evaluated.

## 2. Materials and Methods

This systematic review was conducted according to the Preferred Reporting Items for Systematic Reviews and Meta-Analyses (PRISMA) guidelines [18], according to a protocol published in the PROSPERO database (CRD42021268105).

### 2.1. Eligibility Criteria

Following the PICO model for a systematic review, we selected the studies considering these parameters: patients (P), healthy adult athletes; intervention (I), note dose of prebiotics supplementation; comparison (C), comparison with a placebo group; outcomes (O), prevention of gastrointestinal and respiratory tract infections. Only randomized controlled studies performed on healthy adult athletes with a note dose of probiotics supplementation were included. The inclusion and exclusion criteria adopted for the systematic review are shown in Table 1.

### 2.2. Outcomes

We considered as outcomes the effects of probiotics supplementation on the prevention of gastrointestinal and respiratory tract infections in athletes. Where no changes in the incidence of the aforementioned infections were reported, the effects of probiotics on the severity of symptoms were considered.

### 2.3. Literature Search and Selection of Studies

The search was conducted until 27 July 2021 using the following terms: (“probiotic”) AND (“sport” OR “exercise” OR “athlete” OR “physical activity”). We searched the following electronic databases: (1) MEDLINE (PubMed); (2) Web of Science; (3) Scopus; and 4) SPORTDiscus (EBSCO). Moreover, we manually searched for further articles in the reference lists of available reviews. We did not restrict the search by date or publication status. Only articles in English, Spanish, Italian, and French languages were included.

### 2.4. Data Collection

Two reviewers (M.D.D. and P.C.) independently assessed the results of the electronic search. Duplicate articles were excluded, then potentially eligible studies were identified and selected based on title and abstract. Full texts of the potentially eligible studies were thoroughly reviewed independently by the same reviewers and those studies that met the selection criteria were included in the analysis. Disagreements were resolved by the opinion of another reviewer (V.D.O.).

For each study, the following information was tabulated by another two reviewers (G.C. and C.P.P.): author and year of publication, main aim of the study, sample size and demographic characteristics, type of study design, type, dose, administration route and length of the supplementation, and main results on health and performance.

### 2.5. Risk of Bias

The risk of bias of the studies was assessed using the revised Cochrane Risk-of-Bias tool for randomized trials (RoB2) [19]. The evaluation was performed by two independent reviewers (P.C. and G.C.). Disagreements were resolved by a third reviewer (M.D.D.). The randomization process, deviations from intended interventions, data on missing outcomes, measurement of outcomes, and selection of reported outcomes were reviewed. Based on this assessment, the risk of bias for each study was categorized as low, moderate, or high.

## 3. Results

### 3.1. Study Selection and Characteristics

**Figure 1:**
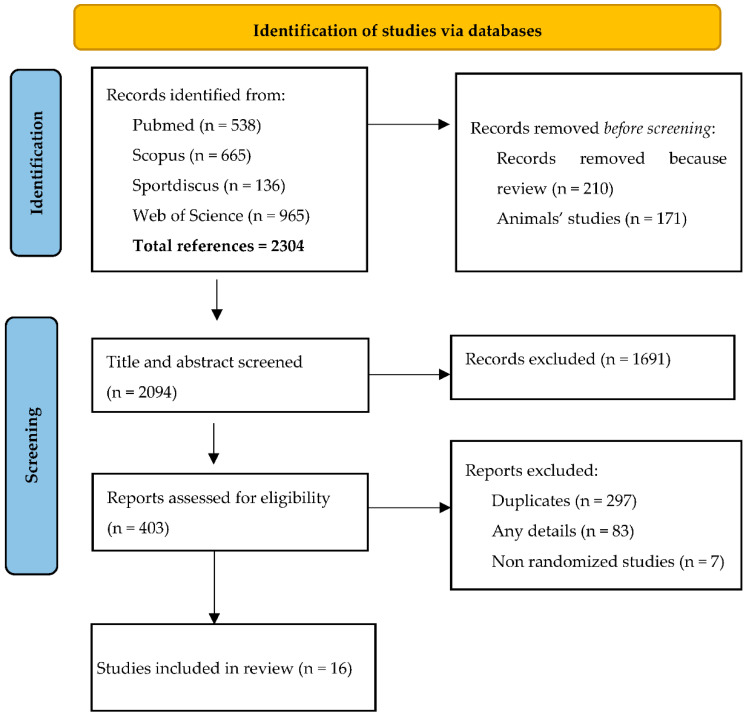
PRISMA flow diagram of the selection of the articles included.

As shown in Figure 1, the primary search identified 2304 relevant articles. After eliminating studies on animal and on unhealthy subjects and reviews, and after the screening of titles and abstracts, 403 studies were considered eligible. From these, duplicates and non-randomized studies were then eliminated and, in accordance with the inclusion and exclusion criteria (Table 1), 16 studies [20,21,22,23,24,25,26,27,28,29,30,31,32,33,34,35] were selected for this systematic review.

The selected studies were published between 2006 and 2021, and they were carried out in different geographic areas: Europe (Austria, UK, Leicestershire, Finland, Serbia), West Asia (Israel), East Asia (Japan), South America (Brazil), and Oceania (New Zealand). All the studies were randomized and the majority of them had a double-blind, placebo-controlled design.

Table 2 shows the sample characteristics for each study. A total of 915 subjects were included and were considered in the analyses of the present review; four studies did not report the participants’ gender [23,24,31,33], seven studies involved only males [20,21,22,26,28,31,34], and five studies included both genders [25,27,29,30,35]. The studies involved adult participants with an average age between 19 and 50 years.

Ten studies concern endurance athletes [20,21,22,24,25,27,30,32,34,35], two studies concern rugby players [26,31], two studies refer to athletes in general [28,33], and two studies involved subjects who played various sports [23,29]. In nine studies [21,22,23,24,25,27,28,29,35], the probiotic supplementation was represented by a single bacterial strain, in the remaining studies a multistrain probiotic was assumed.

Regarding the risk of bias assessment, eleven studies showed a low risk of bias (Table 3).

### 3.2. Outcomes

#### 3.2.1. Single-Strain Probiotics Supplementation

##### Prevention of Respiratory Infections

Eight studies investigated the effects of single bacterial strain probiotic supplementation on the prevention, occurrence, duration, and severity of respiratory tract infections. Cox et al. conducted a trial on a group of healthy elite male distance runners for a duration of 28 days. The supplementation of a probiotic composed of *Lactobacillus fermentum* VRI-003 resulted in a reduction in the number of days of respiratory infection and a tendency to a lesser extent of the disease [21]. Gill et al. evaluated the effects of a short-term (7 days) supplementation of *L. casei* on a group of healthy endurance-trained male runners. They deduced that this kind of supplementation does not provide further immune protection at the level of the respiratory oral mucosa [22]. Gleeson et al. conducted two studies in which they used two different bacterial strains for 16 weeks. In the first study, they administered a probiotic based on *L. casei shirota* to a group of healthy subjects who were engaged in regular sports training (various sports); in this case, there was a reduction in the frequency of respiratory infections correlated with a better maintenance of salivary immunoglobulin A (IgA) levels [23]. In the second study, they conducted the experimentation on a group of endurance athletes by administering them a probiotic based on *L. salivaris*. In this case, there was no reduction in the frequency of respiratory infections, nor did the supplementation affect lymphocyte counts or antimicrobial protein levels [24]. In a further study, Gleeson et al. evaluated the effects of long-term (5 months) supplementation of a probiotic based on *L. casei Shirota* on a group of endurance athletes. This kind of supplementation did not reduce the episodes of respiratory infections, however, it resulted in a reduction in antibodies towards cytomegalovirus and Epstein–Barr virus in people with these infections [25]. Komano et al. found a reduction in cumulative days of respiratory infection symptoms following the short-term (13 days) administration of a probiotic based on *Lactococcus lactis* to a group of healthy male athletes [28]. Marinckovic et al. found only a trend towards a reduction in the severity of respiratory infections, the duration of episodes, and the number of symptoms (*L. helveticus Lafti* L10) [29]. However, West et al. obtained uncertain results following the administration of *L. fermentum* VRI-003 PCC for 11 weeks in a group of agonist cyclists. The burden (duration × severity) of lower respiratory disease symptoms was lower only in males, while it increased in females [35].

##### Prevention of Gastrointestinal Infections

Regarding the possible effects of probiotic supplementation on the prevention of and reduction in the incidence of infections affecting the gastrointestinal tract, two studies have investigated this. Kekkonen et al. found a reduction in the duration of episodes of GI infections following the supplementation of *L. rhamnosus* for a period of 3 months to a group of marathon runners [27]. West et al. found a decrease in the severity of GI disease by administering a probiotic based on *L. fermentum* VRI-002 PCC to a group of agonist cyclists for a period of 11 weeks [35].

#### 3.2.2. Multispecies Probiotics Supplementation

##### Prevention of Respiratory Infections

Five studies have investigated the possible effects of multispecies probiotics on respiratory infections. Only Batatinha et al. found no results on the role of probiotics in the prevention of these diseases (*Bifidobacterium animalis* subsp. *Lactis* and *L. acidophilus* for 30 days) [20]. The other studies show encouraging results. Haywood et al. found no episodes of respiratory infections in elite male rugby union players, following the administration of probiotics for 4 weeks [26]. Pumpa et al. reported a lower incidence of infections in a group of rugby players after a 27-week integration period [31]. Strasser et al., after 3 months of supplementation of *Bifidobacterium bifidum* W23, *Bifidobacterium lactis* W51, *Enterococcus faecium* W54, *L. acidophilus* W22, *L. brevis* W63, and *Lactococcus lactis* W58, noted a limited rate of tryptophan degradation directly related to a lower incidence of respiratory infections in trained athletes [33]. Tavares et al. found a mitigating effect on the incidence of respiratory infections in a group of male marathon runners, deriving from the 30 days administration of *L. acidophilus* LB-G80, *L. paracasei* LPc-G110, *L.* subp. *Lactis* LLL-G25, *B. animalis* subp. *Lactis* BL-G101, and *B. bifidum* BB-G90 [34].

##### Prevention of Gastrointestinal Infections

Four studies investigated the GI tract and all of them showed a beneficial effect of probiotics. Haywood et al. found no episodes of disease [26]. Pugh et al. observed a reduction in the incidence and severity of symptoms associated with GI diseases in a group of marathon runners, after 28 days of administration of *L. acidophilus* (CUL60 and CUL21), *B. bifidum* (CUL20), and *B. animalis* subsp. *Lactis* (CUL34) [30]. Pumpa et al. found a reduction in the onset of GI infections [31]. Schreiber et al., after 90 days of supplementation, noted a reduction in symptoms associated with GI infections in a group of male cyclists supplemented with *L. helveticus Lafti* L10, *B. animalis* ssp. *lactis Lafti* B94, *E. faecium* R0026, *B. longum* R0175, and *Bacillus subtilis* R0179 [32].

## 4. Discussion

The studies included in this review concern experiments conducted from 2006 to 2021 examining the effects of probiotics on athletes in the context of various sports disciplines. In particular, these studies aimed to investigate how the use of probiotics can prevent or alleviate the main infections related to high-intensity exercise.

With regard to disorders affecting the GI tract, the results suggest that both the integration of single-strain probiotics, based mainly on *Lactobacillus* spp., and that of multispecies probiotics produce protective effects against these pathologies, after a long-term administration ranging from a minimum of 4 weeks to a maximum of 27 weeks. With regard to the integration of single-strain probiotics, minimal dosages, compared to the higher ones experienced in the administration of multispecies probiotics, but taken on a long-term basis, are already able to help in reducing the duration of GI infections. However, the use of probiotics based on multispecies probiotics at even lower dosages and fewer times can provide more effective protection. In particular, the association of Bifidobacteria, Lactobacilli, and *E. faecium* is able to reduce the incidence of nausea, belching, and vomiting and of GI symptoms due to intense physical exercise.S

Comparing the effects of single bacterial strains with that of multispecies probiotics, it can be deduced that while the former can reduce the duration of episodes of disease/disorders and the severity of infections, the latter actually have a predominantly preventive action, managing to intervene in intestinal permeability and reducing the incidence, as well as the severity, of symptoms affecting the GI tract.

As for respiratory diseases, the results are encouraging in the case of both single-strain and multispecies probiotics consumption. In particular, the administration of *L. casei Shirota* has been shown to be effective in reducing the frequency of respiratory infections. Short-term administration of *L. lactis* at high doses also resulted in a reduction in cumulative days of respiratory symptoms. *L. fermentum* VRI-003 led to a reduction in the number of days of illness and a tendency to a reduction in the severity of the disease (administration for 28 days, 2 billion), and a reduced symptom burden (given for 11 weeks, 1 billion). On the other hand, no results have been obtained from the administration of *L. salivarius*, albeit in high doses and for a long period, of *L. casei* after a short-term administration (7 days), and of *L. casei Shirota* in another study, which, however, found a low incidence of infections.

With regard to the effects of multispecies probiotics, the combination of various strains of Lactobacilli and Bifidobacteria has determined excellent results in terms of reduction in incidence, further underlining the power of multispecies integration. Only one study did not show any results, which is probably attributable to the poor variety of species administered in the 30 days before the competition. In fact, in another considered study, which provided a lower dose of probiotics (5 billion) and a richer variety of species for an equal time, a mitigation of the incidence of infections was found. So, contrarily to the theory that the use of each bacterium separately can produce results while their combination can be less effective or not at all effective [36], in this case it is precisely the heterogeneity of the probiotics that gave a greater effectiveness.

As regards the type of physical exercise, the most represented one is endurance sports [20,21,22,24,25,27,30,32,34,35] in which the supplementation of probiotics has determined, in most cases, positive effects in terms of mitigation of the symptoms associated with respiratory and gastrointestinal tract infections and, in one study, in terms of reducing the incidence of respiratory [34] infections. In this case, the supplementation concerned multispecies probiotics, further underlining the importance of the association of multiple bacterial species.

While there are only two studies [26,31] involving rugby players in the review, they offer interesting results. In both studies, supplementation included the use of multispecies probiotics following which a reduction in the incidence of gastrointestinal and respiratory infections was recorded.

As for the studies conducted on subjects who performed various types of physical exercise [23,28,29,33], the supplementation with a probiotic based on *L. casei Shirota* produced a reduction in the frequency of URTIs in one study [23]. Furthermore, the supplementation of multispecies probiotics in this case also determined beneficial effects in terms of reducing the incidence of URTI.

As for the formulations used for the administration of probiotics, in most of the studies including probiotics these were administered in capsules including maltodextrin as an additive. Maltodextrin is a polysaccharide, used as a food additive and commonly added as a stabilizer, coating material, or bulking agent. Scientific studies conducted in recent years have concerned the potential effects of the consumption of maltodextrin, not only in animals, but also in humans. In particular, it was shown that in humans the use of maltodextrin represents a stressor for the endoplasmic reticulum of intestinal epithelial cells with a consequent reduction in mucus production downstream, thus determining an increase in susceptibility to colitis [37]. In the case of athletic subjects, intense physical activity causes an increase in intestinal permeability, which makes the intestinal barrier more susceptible to the entry of substances such as toxins and pathogens. So, it should be clarified whether the maltodextrin used in probiotic supplements can somehow negatively affect the beneficial effects of the probiotic itself. All this must also be contextualized with respect to the type of diet followed and, therefore, the overall quantity of maltodextrin taken in the diet.

This systematic review has strengths and limitations. The strengths concern the level of evidence coming from the analyzed studies, which are all randomized and showed a generally high quality. As for the limitations, in the included research, probiotics are administered in different quantities and forms for a variable duration of the intervention. In particular, as underlined in the review, some studies were based on the administration of a single-species probiotic, while others examined the effects of multispecies compounds, which led us to separate the corresponding results. Furthermore, some of the included studies did not use probiotics alone but with other substances, making it difficult to interpret the results.

It is difficult to conceive of a probiotic as primary prevention, and it will probably never be possible to consider its real benefit in secondary prevention. Future research could be useful, for example, in the context of network meta-analyses to analyze the best probiotic formulation, but it is still difficult, not being able to build a reliable control group. Probiotic supplementation has traditionally focused on gut health, however, performance increases that have been underestimated in recent years should be further investigated in the context of possible doping effects [38,39].

## 5. Conclusions

In recent years, the interest in the effects of probiotics on athletes’ health has been growing worldwide. In particular, the evidence coming from the available literature suggests that the integration of the athletes’ diet with certain bacterial strains could be useful to help them to prevent infections affecting the gastrointestinal and respiratory systems, or to reduce the related symptomatology. Since intense physical exercise can increase the risk of developing these infections by lowering the immune defenses of athletes, the perspective of probiotic use in a sport setting is worthy of attention. In order to achieve stronger and more specific evidence on this issue, further research is needed to identify which category of athletes can benefit most by probiotics supplementation, which microbial species can be more effective, and in which circumstance (i.e., climatic conditions, sport season, epidemiological situations, etc.) their use can be recommended.

## Figures and Tables

**Table 1:** Inclusion and exclusion criteria for the examined studies.

Inclusion Criteria	Exclusion Criteria
Studies on healthy adult athletes undergoing probiotics supplementation as intervention Randomized controlled studies Studies reporting the type and dose of probiotics	Studies on animal models Studies on individuals with pathological conditions Studies on non-active individuals Studies on children

**Table 2:** Characteristics of the included studies.

Author, Year, Country, Study Design	Sample Characteristics N^o^ of Subjects, M/F, Mean Age	Probiotics/Prebiotics Daily Intake Intervention Length	Main Results
Batatinha, 2020 Brazil -randomized placebo-controlled double-blind study	Male marathonists 27 subjects PRO (35.96 ± 5.81) PLA (40.46 ± 7.79)	(*Bifidobacterium*-*animalis*-subsp.-*Lactis* (10 × 10^9^) and *Lactobacillus*-*Acidophilus* (10 × 10^9^) + 5 g of maltodextrin). PLA: only maltodextrin 30 days	The total number of CD8 T cells and the memory subsets statistically decreased only in the placebo group post-race. Pro-inflammatory cytokine production by stimulated lymphocytes decreased in the probiotic group after the supplementation period. Data from the URTI questionnaire showed that both placebo and probiotic group presented low level of URTI with no statistical difference between the groups.
Cox, 2007 Australia -double-blind, placebo-controlled, crossover design	Healthy elite male distance runners N = 20 27.3 ± 6.4 years	*L. fermentum* VRI-003 (PCC), contained a minimum of two billion of *L. fermentum* strain VRI-003 viable organisms in hard gelatin capsules with microcrystalline cellulose as the excipient PLA: only microcrystalline cellulose 28 days	Significant reduction in the number of days of respiratory illness symptoms, and a trend towards a lower severity of illness, during *L. fermentum* VRI-003 treatment compared with placebo.
Gill, 2016 UK -blinded, randomized, placebo-controlled	Healthy endurance-trained male runners N = 8 26 ± 6 years	Probiotic beverage containing *L. casei* (PRO; 1.0 × 10^11^·day^−1^) 7 days	Daily oral supplementation of a commercially available probiotic beverage containing *L. casei* for 7 consecutive days does not influence S-AMP responses and subsequent oral–respiratory mucosal immune status above placebo in response to EHS in an endurance-trained population
Gleeson, 2011 UK -double-blind, randomized, placebo-controlled	Healthy subjects who were engaged in regular sports training (predominantly endurance-based activities such as running, cycling, swimming, triathlon, team games, and racquet sports) probiotic subjects (n = 32) age 32 ± 14 years placebo subjects (n = 26) age 25 ± 9 years	*L. casei Shirota* (LcS). The probiotic drink contained a minimum of 6.5 × 10^9^ live cells of LcS in each pot 16 weeks	Regular ingestion of LcS appears to be beneficial in reducing the frequency of URTI in an athletic cohort, which may be related to better maintenance of saliva IgA levels during a winter period of training and competition.
Gleeson, 2012 UK -double-blind, randomized, placebo-controlled	Endurance athletes PRO (n = 27) age 25 ± 5 years PLA (n = 27) age 24 ± 4 years	0.2 g of *L. salivarius* (2 × 10^10^ bacterial colony-forming units), with 1.78 g maltodextrin and 0.01 g magnesium stearate. The placebo contained only the same amount of chemical vehicle (maltodextrin and magnesium stearate) 16 weeks	Regular ingestion of *L. salivarius* does not appear to be beneficial in reducing the frequency of URTI in an athletic cohort and does not affect blood leukocyte counts or levels of salivary antimicrobial proteins during a spring period of training and competition.
Gleeson, 2016 Leicestershire -randomized controlled trial	Endurance athletes 268 subjects 113 (42%) females 155 (58%) males 21 ± 3 years (mean ± SD)	Drink contained a minimum of 6.5 × 10^9^ live cells of *L. casei Shirota* (twice per day) 5 months	A significant time × group interaction effect was observed for plasma CMV antibody titers in CMV seropositive participants (*p* < 0.01) with antibody titer falling in the PRO group but remaining unchanged in the PLA group over time. A similar effect was found for plasma EBV antibody titers in EBV seropositive participants (*p* < 0.01) with antibody titer falling in the PRO group but increasing in the PLA group over time. In summary, regular ingestion of PRO did not reduce URS episode incidence which might be attributable to the low URS incidence in this study. Regular ingestion of PRO reduced plasma CMV and EBV antibody titers, an effect that can be interpreted as a benefit to overall immune status.
Haywood, 2013 New Zealand -randomized control trial with two arms: placebo and probiotic	Elite male rugby union players Thirty elite rugby union players; 24.7 ± 3.6 years old	Three strains of bacteria (*L. gasseri*: 2.6 billion colony-forming units (CFU), *Bifidobacterium bifidum*: 0.2 billion organisms, *Bifidobacterium longum*: 0.2 billion organisms) 4 weeks	During the probiotic treatment, 14/30 participants never experienced a single upper respiratory tract illness (URTI) or gastrointestinal (GI) episode, compared to 6/30 on the placebo supplementation (*p*= 0.033). The mean number of days of illness tended to be higher for the placebo (5.8 ± 6.6 days) than probiotic (3.4 ± 4.6 days) (*p* = 0.054).
Kekkonen, 2007 Finland -randomized, double-blind, placebo-controlled, parallel group	Marathon runners LGG, n = 70; 62 M/8 F; age 40 (22–58) Placebo, n = 71; 63 M/8 F; age 40 (23–69)	*L. rhamnosus* GG; two LGG bottles provided a total of 4 × 10^10^ bacteria or 2 capsules per day—a daily total of 1 × 10^10^ LGG bacteria -3 months	There were no differences in the number of respiratory infections or GI-symptom episodes. The duration of GI-symptom episodes in the LGG group was 2.9 vs. 4.3 d in the placebo group during the training period (*p* = 0.35) and 1.0 vs. 2.3 d, respectively, during the 2 weeks after the marathon (*p* = 0.046). LGG had no effect on the incidence of respiratory infections or GI-symptom episodes in marathon runners, but it seemed to shorten the duration of GI-symptom episodes.
Komano, 2018 Japan -randomized, placebo-controlled, double-blinded trial	Healthy male athletes Placebo (n = 24) 20.5 ± 0.8 LC-Plasma (n = 26) 20.8 ± 0.8	LC-Plasma capsules containing 100 billion cells of heat-killed *Lactococcus lactis* strain plasma and cornstarch 13 days	CD86 as maturation marker on pDC was significantly increased in the LC-Plasma group. Cumulative days of URTI were significantly lower in the LC-Plasma group and symptoms such as sneezing or running nose were significantly lower in the LC-Plasma group. Moreover, the cumulative days of fatigue were significantly fewer in the LC-Plasma group.
Marinkovic, 2016 Serbia -randomized, double-blind, placebo-controlled trial	Elite athletes (badminton, triathlon, bicycling, athletics, karate, kayaking, and judo) Probiotic n = 20; Males/females 15/5; age (years) 23.5 ± 2.7 Placebo n = 19; Males/females 14/5; age (years) 22.8 ± 2.5	Probiotic capsules of *L. helveticus Lafti* L10 (2 × 10^10^ CFU) -capsules contained 72.2% of the bacterial mass, 26.7% maltodextrin, and 1% magnesium stearate. -placebo capsules contained 1% magnesium stearate and 99% maltodextrin 14 weeks	Neither the incidence nor the severity of respiratory infection differed between the treatments, although a trend for decreasing severity in the probiotic group emerged (*p* = 0.078). However, the duration of a URTI episode was shorter in the probiotic group than in the placebo group (7.25 ± 2.90 versus 10.64 ± 4.67 days). Moreover, there were fewer reported symptoms of URTI in the probiotic group.
Pugh, 2019 UK -double-blind, randomized, and matched-pairs design	Recreational runners 24 (20 M/4 F); PRO group 34.8 ± 6.9	Capsule (25 billion CFU] *L. acidophilus* (CUL60 and CUL21), *Bifidobacterium bifidum* (CUL20), and *Bifidobacterium animalis* subsp. *Lactis* (CUL34)) -28 days	Prevalence of moderate GI symptoms was lower during the 3/4 weeks of the supplementation period compared to the first and second weeks in PRO (*p* < 0.05) but not PLC (*p* > 0.05). During the marathon, GI-symptom severity during the final third was significantly lower in PRO compared to PLC (*p* = 0.010). Circulatory measures increased to a similar extent between PRO and PLC (*p* > 0.05).
Pumpa, 2019 Australia -double-blind, randomized, controlled trial	Elite rugby union athletes Probiotic (n = 9) mean age 27.03 ± 3.15 years Placebo (n = 10) mean age 26.56 ± 2.87 years	60 billion viable bacteria *L. rhamnosus, L. casei*, *L. acidophilus*, *L. plantarum*, *L. fermentum*, *Bifidobacterium lactis*, *Bifidobacterium bifidum*, *Streptococcus thermophilus* The second probiotic supplement which was consumed in conjunction with the Ultrabiotic 60™ during the international competition phase was SBFloractiv ™ (FIT-BioCeuticals Ltd., Alexandria, Australia) and contained 250 mg *Saccharomyces boulardi* 27 weeks	The probiotic protocol used in this study was associated with an increase in salivary alpha-amylase, supporting its possible role as a host defense peptide.
Schreiber, 2021 Israel -randomized, double-blind, two-arm, placebo-controlled trial design	Male cyclists, ranked elite or category 1 level competitions Multistrain probiotic-supplemented group (E, n = 11) 25.9 ± 4.6 Placebo group (C, n = 16) 29.5 ± 6.2	The probiotic supplement contained about 15 billion CFU of a probiotic blend consisting of 5 strains: at least (≥) 4.3 × 10^9^ CFU *L. helveticus Lafti* L10 (28.6%), ≥4.3 × 10^9^ CFU *Bifidobacterium animalis* ssp. *lactis Lafti* B94 (28.6%), ≥3.9 × 10^9^ CFU *Enterococcus faecium* R0026 (25.7%), ≥2.1 × 10^9^ CFU *Bifidobacterium longum* R0175 (14.3 %), and ≥0.4 × 10^9^ CFU *Bacillus subtilis* R0179 (2.8%) 90 days	Lower incidence of nausea, belching, and vomiting (*p* < 0.05) at rest, and decreased incidence of GI symptoms during training were found in E group vs. C group (ΔGI −0.27 ± 0.47% vs. 0.08 ± 0.29%, *p* = 0.03), no significant changes were observed in the incidence of total overall GI symptoms (ΔGI −5.6 ± 14.7% vs. 2.6 ± 11.6%, *p* = 0.602). Mean rate of perceived exertion (RPE) values during the TTF were lower in E group (ΔRPE: −0.3 ± 0.9 vs. 0.8 ± 1.5, *p* = 0.04).
Strasser, 2016 Austria -randomized, double-blinded, placebo-controlled trial	Trained athletes Probiotics (n = 14) 25.7 ± 3.5 Placebo (n = 15) 26.6 ± 3.5	Multispecies probiotics composed of six strains consisting of *Bifidobacterium bifidum* W23, *Bifidobacterium lactis* W51, *Enterococcus faecium* W54, *L. acidophilus* W22, *L. brevis* W63, and *Lactococcus lactis* W58 (Ecologic^®^ Performance, Winclove B.V., Amsterdam, the Netherlands). The total cell count was adjusted to 2.5 × 10^9^ colony forming units (CFU) per gram. The matrix consisting of cornstarch, maltodextrin, vegetable protein, MgSO_4_, MnSO_4_, and KCl. Subjects were instructed to take 1 sachet of 4 g per day, which is equivalent to 1 × 10^10^ CFU/day 3 months	Data indicate reduced exercise-induced tryptophan degradation rates in the PRO group. Daily supplementation with probiotics limited exercise-induced drops in tryptophan levels and reduced the incidence of URTI, however, it did not benefit athletic performance.
Tavares-Silva, 2021 Brazil -randomized, double-blind, placebo study	Marathon runners, males Probiotic group (n = 7); age (years) 41.57 ± 3.20 Placebo group (n = 7); age (years) 38.28 ± 3.09	5 billion colony-forming units (5 × 10^9^ CFU) of a multistrain probiotic, consisting of 1 billion CFU of each of *L. acidophilus* LB-G80, *L. paracasei* LPc-G110, *Lactococcus* subp. *lactis* LLL-G25, *Bifidobacterium animalis* subp. *lactis* BL-G101, and *Bifidobacterium bifidum* BB-G90 30 days	Despite the low number of marathoners participating in the study, probiotic supplementation showed a capability to preserve the functionality of monocytes and mitigate the incidence of URTI.
West, 2011 Australia -randomized control trial	Competitive cyclists 87 subjects (62 M/35 F) PRO group 29/18 (M 35.2 ± 10.3) (F36.5 ± 8.6) PLA group 33/17 (M 36.4 ± 8.9) (F 35.6 ± 10.2)	Probiotic capsule contained a minimum of one billion (10^9^) colony-forming units of *L. fermentum* VRI-003 PCC^®^ (Probiomics Ltd., Sydney, Australia) 11 weeks	There was a substantial 0.7 (0.2 to 1.2) scale step reduction in the severity of gastrointestinal illness at the mean training load in males, which became more pronounced as training load increased. The load (duration × severity) of lower respiratory illness symptoms was lower by a factor of 0.31 (99%CI; 0.07 to 0.96) in males taking the probiotic compared with placebo but increased by a factor of 2.2 (0.41 to 27) in females.

**Table 3:** Methodological quality of the studies using the tool RoB 2.0.

First Author Name	Randomization Process	Deviation from the Intended Intervention	Missing Results Data	The Measurement Result	Selection of the Result Reported	General Trend
Batatinha, 2020	Low	Low	Low	Low	Low	Low
Cox, 2007	Low	Low	Low	Low	Low	Low
Gill, 2016	Low	Low	Low	Low	Low	Low
Gleeson, 2011	Low	Low	Low	Low	Low	Low
Gleeson, 2012	Low	Low	Low	Low	Low	Low
Gleeson, 2016	Low	Low	Low	Low	Low	Low
Haywood, 2013	Some concerns	Some concerns	Low	Low	Some concerns	High
Kekkonen, 2007	High	Low	Low	Highs	Low	High
Komano, 2018	Low	Low	Low	High	Low	High
Marinkovic, 2016	Low	Low	Low	High	Low	High
Pugh, 2019	Low	Low	Low	Low	Low	Low
Pumpa, 2019	Low	Low	Low	Low	Low	Low
Schreiber, 2021	Some concerns	Low	Some concerns	Low	Low	High
Strasser, 2016	Low	Low	Low	Low	Low	Low
Tavares-Silva, 2021	Low	Low	Low	Low	Low	Low
West, 2011	Low	Low	Low	Low	Low	Low

## Data Availability

Not applicable.
